# “Facekit”—Toward an Automated Facial Analysis App Using a Machine Learning–Derived Facial Recognition Algorithm

**DOI:** 10.1177/22925503211073843

**Published:** 2022-01-24

**Authors:** Omri Nachmani, Tomas Saun, Minh Huynh, Christopher R. Forrest, Mark McRae

**Affiliations:** 1 Michael G. DeGroote School of Medicine, 3710 McMaster University, Hamilton, Ontario, Canada; 2 Faculty of Medicine, 12366 University of Toronto, Toronto, Ontario, Canada; 3 Division of Plastic and Reconstructive Surgery, 3710 McMaster University, Hamilton, Ontario, Canada

**Keywords:** machine learning, facial recognition, facial analysis, cosmetic, facial cannons

## Abstract

**Introduction:** Multiple tools have been developed for facial feature measurements and analysis using facial recognition machine learning techniques. However, several challenges remain before these will be useful in the clinical context for reconstructive and aesthetic plastic surgery. Smartphone-based applications utilizing open-access machine learning tools can be rapidly developed, deployed, and tested for use in clinical settings. This research compares a smartphone-based facial recognition algorithm to direct and digital measurement performance for use in facial analysis. **Methods:** Facekit is a camera application developed for Android that utilizes ML Kit, an open-access computer vision Application Programing Interface developed by Google. Using the facial landmark module, we measured 4 facial proportions in 15 healthy subjects and compared them to direct surface and digital measurements using intraclass correlation (ICC) and Pearson correlation. **Results:** Measurement of the naso-facial proportion achieved the highest ICC of 0.321, where ICC > 0.75 is considered an excellent agreement between methods. Repeated measures analysis of variance of proportion measurements between ML Kit, direct and digital methods, were significantly different (*F*[2,14] = 6-26, *P*<<.05). Facekit measurements of orbital, orbitonasal, naso-oral, and naso-facial ratios had overall low correlation and agreement to both direct and digital measurements (*R*<<0.5, ICC<<0.75). **Conclusion:** Facekit is a smartphone camera application for rapid facial feature analysis. Agreement between Facekit's machine learning measurements and direct and digital measurements was low. We conclude that the chosen pretrained facial recognition software is not accurate enough for conducting a clinically useful facial analysis. Custom models trained on accurate and clinically relevant landmarks may provide better performance.

## Résumé

**Introduction :** Il existe de multiples outils pour procéder aux mesures et à l’analyse des caractéristiques faciales à l’aide des techniques d’apprentissage machine de la reconnaissance faciale. Cependant, il reste plusieurs défis à relever avant qu’ils soient utiles en contexte clinique de chirurgie reconstructive et de chirurgie plastique. Des applications pour téléphone intelligent faisant appel à des outils d’apprentissage machine en libre accès peuvent être rapidement mises au point, déployées et mises à l’essai dans un cadre clinique. Dans la présente étude, les chercheurs comparent un algorithme de reconnaissance faciale sur téléphone intelligent pour effectuer les mesures directes et numériques nécessaires lors de l’analyse faciale. **Méthodologie :** Facekit est une application pour appareil photo de téléphone Android qui fait appel à ML Kit, une application de vision par ordinateur en libre accès créée par Google. Au moyen du module de repères faciaux, les chercheurs ont mesuré quatre proportions faciales chez 15 sujets en santé et les ont comparées aux mesures de surface directe et aux mesures numériques à l’aide de la corrélation intraclasse et de la corrélation de Pearson. **Résultats :** La mesure de la proportion nasofaciale a obtenu le coefficient de corrélation intraclasse (CCI) le plus élevé, à 0,321, où un CCI supérieur à 0,75 est considéré comme une excellente corrélation entre les méthodes. Des analyses de variance répétées des mesures de proportion entre le ML Kit, la méthode directe et la méthode numérique différaient considérablement (F[2,14] = 6 à 26, *p*<<0,05). Les mesures Facekit des ratios entre les mesures orbitale, orbitonasale, naso-orale et naso-faciale avaient une faible corrélation globale et étaient corrélées avec les mesures directes et numériques (R<<0,5, CCI<<0,75). **Conclusion :** Facekit est une application pour appareil photo de téléphone intelligent visant à analyser rapidement les caractéristiques faciales. La concordance entre les mesures d’apprentissage machine de Facekit et les mesures directes et numériques était faible. Les chercheurs concluent que le logiciel de reconnaissance faciale préentraîné n’est pas assez précis pour procéder à une analyse faciale utile sur le plan clinique. Des modèles personnalisés formés à des repères précis et pertinents sur le plan clinique donneront peut-être un meilleur rendement.

## Introduction

Facial analysis is a fundamental component of any plastic, reconstructive, or maxillofacial consultation. Years of training and experience enable plastic surgeons to rapidly analyze an individual's face and quickly determine an impression of facial pathology, dysmorphism, and beauty. Much like the common proverb: “beauty is in the eye of the beholder,” individual plastic surgeons may have varying ideals when it comes to facial analysis—thus making this form of “gestalt” analysis truly subjective in nature.

The objective measurement of facial landmarks and proportions has become increasingly used to highlight areas of interest in preoperative assessments, detect post-operative changes in facial reanimation or cosmetic surgery, provide an objective measurement tool for researchers, or assist with the diagnosis of various cranial dysmorphologies.^1,2^ The ability to accurately extract facial landmarks and subsequently precisely calculate various proportions is integral to successful facial analysis. Multiple objective techniques for the measurement of soft tissue facial landmark measurements have been described and validated in prior literature.^1,3,4^ Direct surface measurements, popularized and standardized by Farkas, have yielded an extensive list of facial indices and population averages across ethnicities.^1,5–7^ Direct measurements, however, are operator dependent, time-consuming, subject to human error and require close, in-person contact with patients. Advances in photography and computer software have enabled highly accurate capture of human faces using precise markup tools, 3-dimensional reconstruction, and incorporation of computed tomography (CT) cephalometric imaging.^4,8–11^ Although powerful, these techniques are limited by their center-dependent availability, cost, and time.

Machine learning (ML)-derived facial recognition algorithms have increasingly been proven useful for automatic feature extraction, objective beauty measurements, and the diagnosis and management of craniofacial dysmorphology.^12–14^ Unfortunately, most ML tools rarely progress beyond initial prototype development and clinical translation to wide adoption and deployment are not typically seen.^15^ Thus, there remains a need for a fast, cost-effective, scalable, and accurate tool for the detection and analysis of human facial landmarks in clinical settings to serve as a reliable foundation on which to build these valuable and powerful tools.

Facial recognition technology is becoming ever more prevalent and accessible on everyday devices. Recently, Google has published a collection of open-access computer vision models (ML Kit) for deployment in phone-based applications.^16^ ML Kit offers a fast, powerful, easy-to-use pretrained face detection model that was developed by experts in the field of computer vision. This model is capable of real-time facial landmark recognition and provides 133 coordinate points representing the eyes, eyebrows, nose, cheeks, mouth, and facial outline. This open-source Application Programing Interface (API) offers novel opportunities for wider development and deployment of automated tools for facial analysis.

The purpose of this study is to build a proof-of-concept smartphone-based camera application leveraging the ML Kit facial landmark detector for basic facial analysis. We were also interested in the algorithm's ability to detect cranial dysmorphology. We developed an android based phone app, “Facekit,” available for download in the Google Play Store for compatible devices. The app first guides users to capture optimal frontal images of the face and then automatically extracts key facial landmarks and calculates a common set of facial proportions for automated facial analysis. Our goal was to assess the accuracy and utility of this pretrained ML model in measuring facial proportions compared to digital and direct surface measurements. Our app provides a platform for rapid scalability and incremental refinement of an easy-to-use clinical tool for facial analysis.

## Methods

A smartphone camera app was developed for Android devices that leverage ML Kit for facial landmark extraction. It was optimized for clinical use by guiding image capture to a standardized frontal view of faces. Automated measurements of 4 horizontal proportions were calculated and compared to direct surface and digital measurements from 15 healthy volunteers.

### App Development in Android Studio

**Figure 1.**
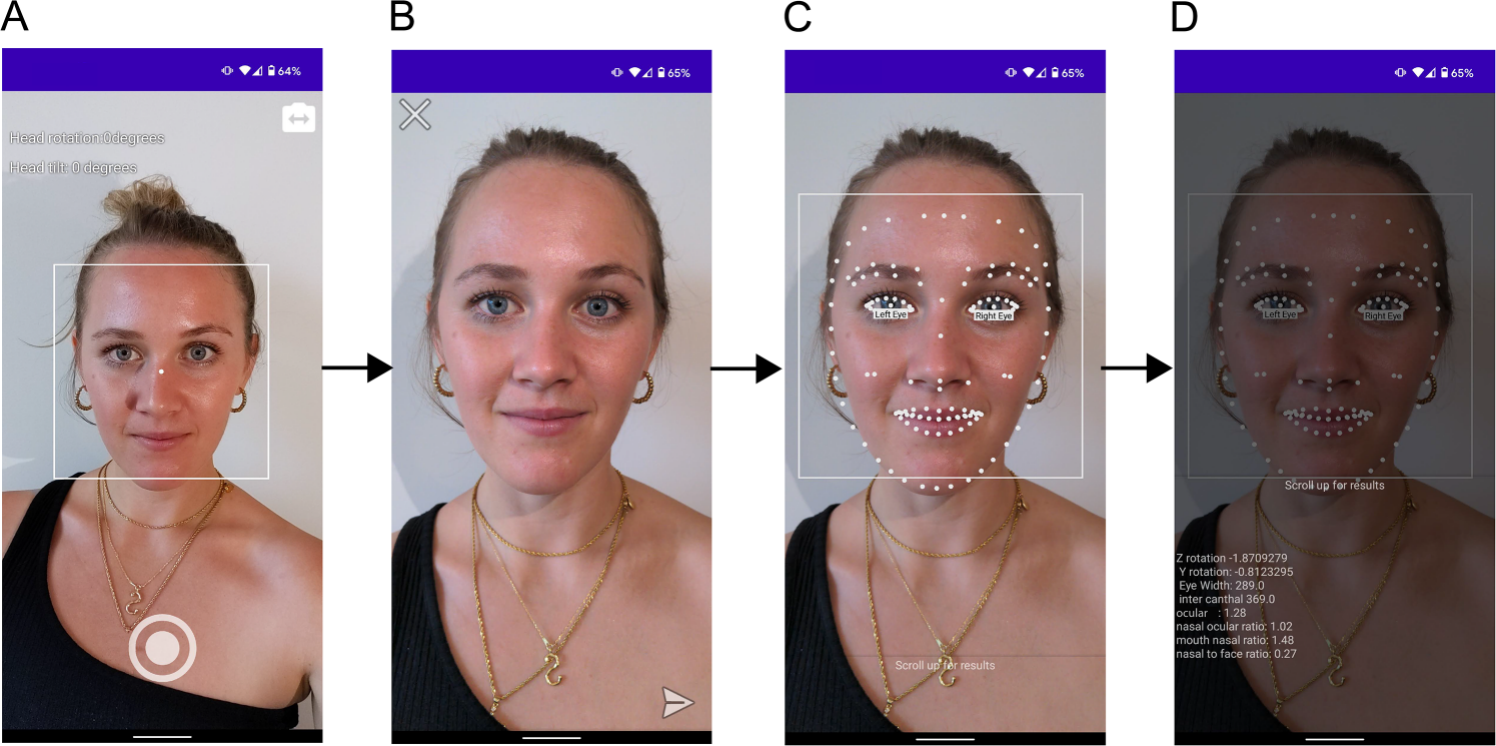
Facekit App workflow. (A) Camera preview provides users with options of using front or back camera, real-time face detection to guide frontal view capture of a consistent image by displaying overhead rotation indicators, (B) Image review phase before analysis, (C) Graphic overlay of facial landmarks post machine learning (ML) processing, and (D) Facial proportion analysis output made visible with a sliding panel. Volunteer images were published with written consent.

For machine learning models to be easily deployable in clinical settings, they must be accessible through phone or computer-based applications that have access to patient data. The Android application, Facekit, was developed for this project using Android Studio in Kotlin programming language (JetBrains Inc) and deployed on a Google Pixel 5 (Google LLC) phone with a 12MP back-camera resolution.^17^ Facekit is a camera-based application capable of detecting and analyzing landmarks of human faces for analysis. To control face image consistency, real-time face detection is activated in the camera preview that displays a bounding box around the face as well as head rotation and tilt information (Figure 1A). The app is programmed to only enable photo capture if head tilt and rotation are under 5°, ensuring consistent capture of frontal views. Prior to processing, users can choose to recapture the image or proceed with the analysis. Facial landmark detection is carried out by the Google ML Kit API (Figure 1C). By swiping up, users have access to basic precalculated facial proportions (Figure 1D).

### Face Detection Model

ML Kit is a mobile SDK developed by Google engineers that provide best-in-class machine learning models for on-device deployment in both Android and iOS apps. This API allows researchers and clinicians to utilize premade computer vision models that would otherwise take too much time, compute power, or human resources to train. Input data processed by this API happens on-device and is not sent to any Google or cloud-based servers. The face detection module can detect human faces, identify key facial features, and get the contours of facial landmarks. The output is a list of Cartesian pixel coordinates representing several facial landmarks that are used in the calculation of facial proportions. Since true length calculations require a pixel to centimeter conversion for each image, the values compared in this study are ratios of lengths instead of the lengths themselves.

### Data Collection

**Figure 2.**
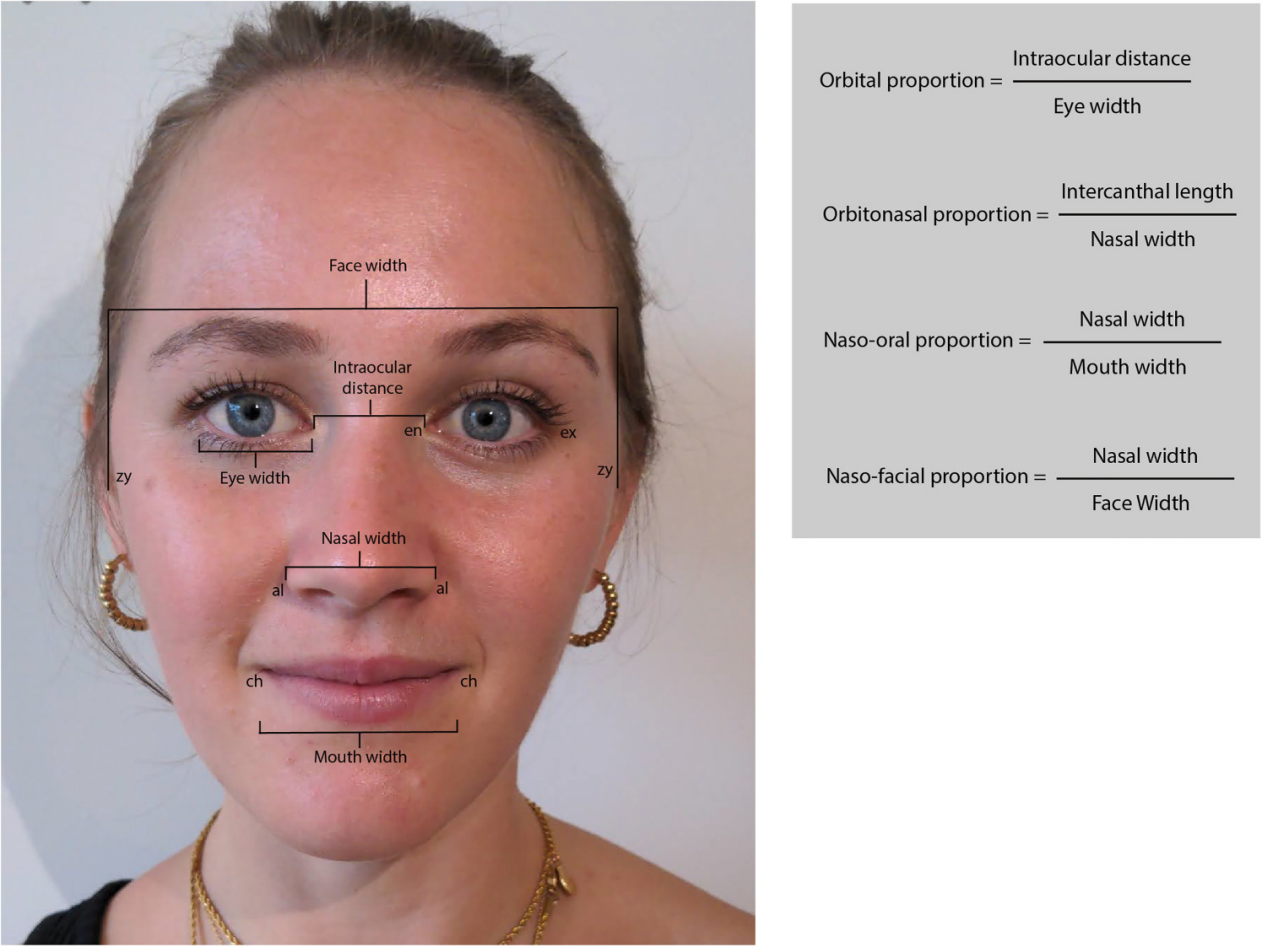
Facial measurements and proportions. Eye width is measured between the exocanthion (ex) and the endocanthion (en). Intercanthal distance is measured between the right and left endocanthions. Nasal width is measured between the 2 alar curvatures (al). Mouth width is measured between the 2 cheilions (ch). Face width is measured between the 2 zygions (zy). Image published with written consent.

All procedures followed were in accordance with the Helsinki Declaration of 1975 as revised in 2008. We recruited 15 healthy participants (8 female; age range 22-30) with diverse ethnic backgrounds and skin tones, and with no identified facial deformities. Participants provided informed verbal consent to be included in this study. Additional written consent was obtained from one participant for whom identifying information is included in this article. In this study, we focused on 4 neoclassical canons of facial proportions which have shown consistency across ethnic backgrounds: orbital (∼1), orbitonasal (∼1), naso-oral (∼1.5), and naso-facial (∼0.25) (Figure 2).^6^ These chosen proportions were considered easily measurable based on digital photographs and the algorithm's output. Direct surface measurements were taken by one author (ON). The landmarks measured were as follows: eye width (ex-en), intraocular distance (en-en), nose width (al-al), mouth width (ch-ch), and face width (zy-zy) (Figure 2). Subsequently, the subject's face was photographed using the Facekit app in frontal view. Subjects sat in a relaxed position with hair tied back and all had minimal or no makeup. Images were saved to the device prior to facial landmark analysis. Once processed, the output of the algorithm was recorded. Digital measurements of the same input image used for the algorithm were performed using Adobe Illustrator (Adobe Inc, 2018, USA) by calculating pixel distances between landmarks using the built-in ruler tool.

### Statistical Analysis

Statistical analysis was performed using Excel (Microsoft Inc) and the Real-Statistics resource pack software (Release 7.6, Zaiontz 2021). The mean and standard deviations of errors between measurements were calculated between direct-ML, digital-ML, and direct-digital methods. One factor repeated measures analysis of variance (ANOVA) (RMA) was used to assess significant differences between the measurement methods. The reliability of each of the ML measurements was assessed by computing the intraclass correlation (ICC) between ML and either direct surface or digital measurements. An ICC of 0.75 or above is considered excellent, between 0.4 and 0.5 is fair/moderate, and below 0.4 is poor. Pearson correlation coefficients were also calculated between each of the measurement techniques. By calculating limits of agreement using the mean and standard deviations, Bland-Altman plots provided a quantitative estimate of how closely measurements from 2 different methods lie and reveal any hidden systemic bias.

## Results

The average measurement error between direct surface and digital methods compared to the ML method is shown in Table 1. Errors between direct and digital measurements were on average smaller than errors involving the ML method (Table 1, top). For methods that measure the same property, we expect their mean errors to not be significantly different. As calculated by repeated measures ANOVA (RMA), the mean errors for the 4 orbital proportions were significantly different in a 3-way comparison of our measurement methods (Table 1, bottom). Notably, the ML method poorly estimates the intraocular distance used in the calculation of orbital and orbitonasal proportion resulting in larger errors compared to other proportions.

**Table 1.** Mean Error and Standard Deviation Between Direct Surface, Digital, and ML Measurements.

		Orbital	Orbitonasal	Naso-oral	Nasal-Facial
Direct—Digital	Mean error	−0.118	0.003	0.110	−0.049
	Standard deviation	−0.122	0.094	0.115	0.023
Direct—ML	Mean error	−0.325	−0.149	−0.006	−0.014
	Standard deviation	−0.167	0.103	0.1700	0.023
Digital—ML	Mean error	−0.207	−0.152	−0.116	0.035
	Standard deviation	−0.226	0.123	0.139	0.033
RMA	*F*	25.9621	19.5938	6.2761	26.6573
	*P* value	4.1948 × 10^−7^	4.766 × 10^−6^	.0055977	3.295 × 10^−7^

Abbreviations: ML, machine learning; RMA, repeated measures ANOVA.

Measurements made by direct, digital, and ML methods were compared using a repeated measured analysis of variance (ANOVA) analysis (RMA) with α set to .05.

**Figure 3.**
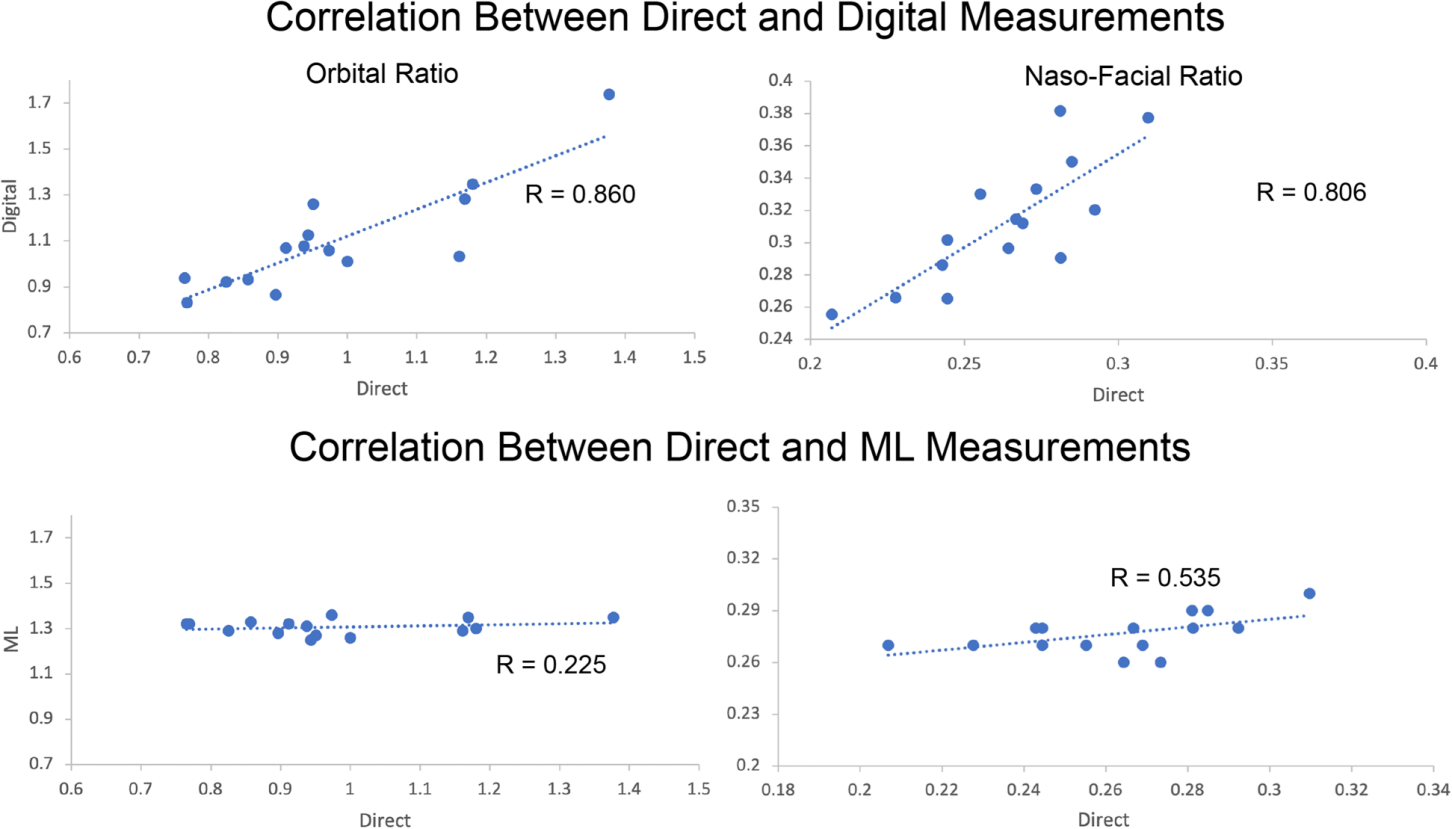
Top: Correlation of orbital ratio between direct surface and machine learning (ML) measurements. Each data point represents one subject. The axis represents the indicated facial proportion as measured by either direct surface or ML methods. Bottom: Correlation of naso-facial ratio between direct-ML measurements (Digital-ML comparisons exhibits a similar lack of correlation and are not shown).

The correlation coefficient (R), ICCs, and 95% confidence intervals for both moth methods against the novel ML methods are listed in Table 2. Comparisons between direct surface and digital measurements are not shown as they have been previously described in the literature. Correlation was high between direct surface and digital measurements for all 4 facial proportions measured (Figure 3, top). Correlation of measurements between the direct surface and ML method was weakest with orbital ratio (r = 0.225) and strongest in nasal-facial ratio (r = 0.535) as shown in Figure 3 bottom panel.

**Table 2.** Correlation (R) and Intraclass Correlation (ICC) Values of All Proportions for Direct Versus ML Measurements and Digital Versus ML Measurements.

		Orbital	Orbitonasal	Naso-oral	Naso-Facial
Direct versus ML	*R*	0.225	0.263	0.280	0.535
	ICC	0.019	0.077	0.156	0.321
	95% CI	−0.068 to 0.211	−0.105 to 0.382	−0.414 to 0.617	−0.118 to 0.687
Digital versus ML	R	0.263	0.110	0.188	0.541
	ICC	0.041	0.03	0.077	0.167
	95% CI	−0.203 to 0.405	−0.138 to 0332	−0.207 to 0.457	−0.135 to 0.536

Abbreviation: ML, machine learning.

**Figure 4.**
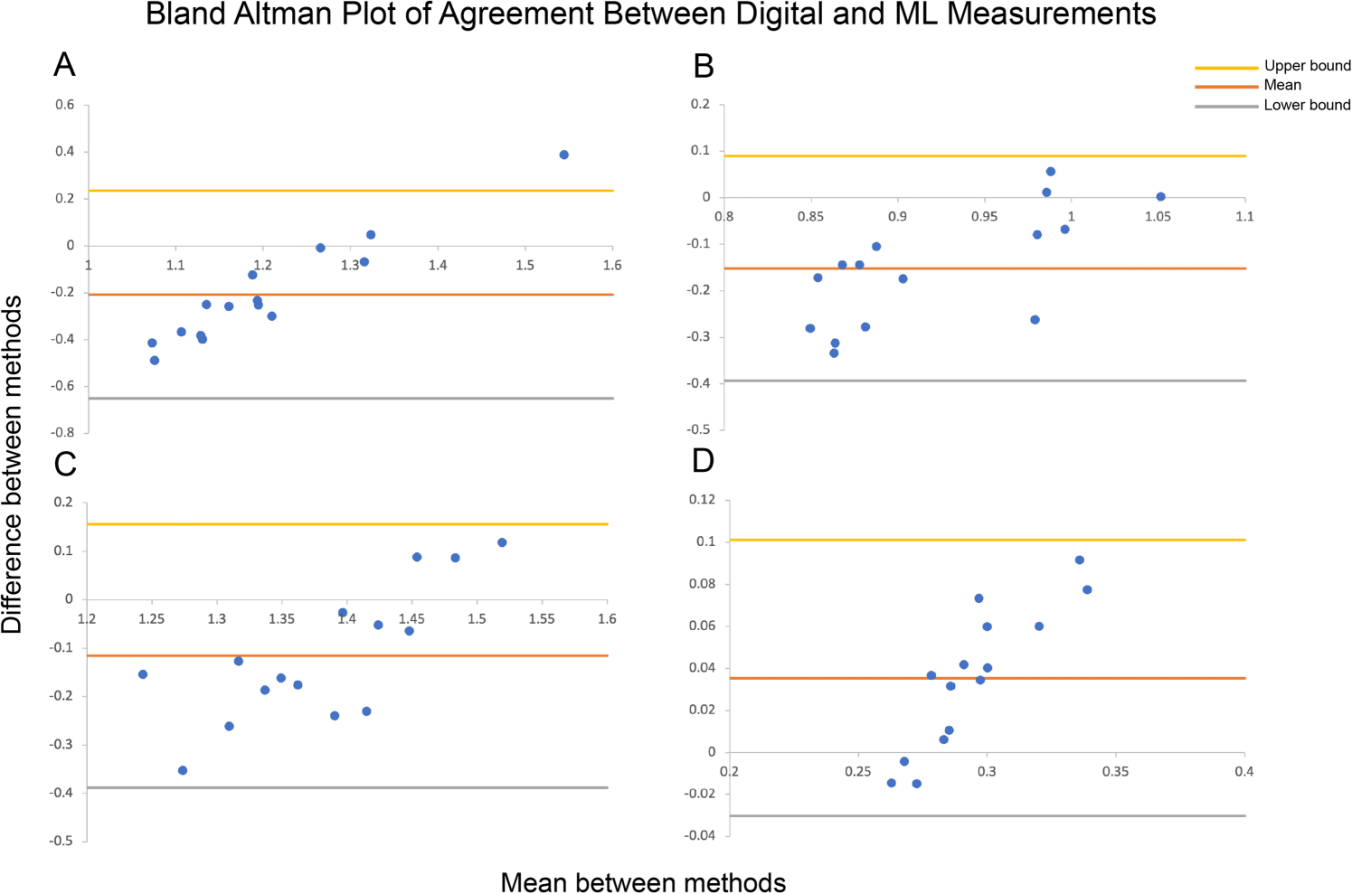
Bland-Altman plots of agreement between digital and machine learning (ML) measurements with lower and upper limits of agreement for (A) Orbital ratio, (B) Orbitonasal ratio, (C) Naso-oral ratio, and (D) Nasal-facial ratio (Direct-ML Bland-Altman plots not shown). Methods with high agreement cluster around the mean, while those with bias or high error will have a larger spread.

None of the facial proportions calculated by ML reached an ICC of 0.75. The naso-facial proportion ICC had the highest upper confidence interval of 0.687. Bland-Altman plots of agreement between digital and ML measurements with lower and upper limits of agreement lines are shown in Figure 4. Bland-Altman plots, or difference plots, evaluate the agreement between 2 methods that measure the same property, since correlation does not necessarily indicate agreement. Methods with high agreement cluster around the mean with minimal spread, while methods with bias or poor accuracy exhibit a larger spread away from the mean. As shown in Figure 4, the data do not cluster around the mean and are spread out across the limits of agreement.

Lastly, the app was tested on publicly available images of craniofacial syndromes that characteristically exhibit hypertelorism: Frontonasal dysplasia, Apert's syndrome, and Crouzon syndrome. ML was unable to correctly identify asymmetrical or disproportionate orbital anatomy and produced orbital ratio measurements within similar ranges to healthy subjects. Facekit was also tested on publicly available images of patients with facial paralysis and was unable to identify the appropriate facial landmarks.

## Discussion

We compared the agreement in measurements of 4 neoclassical facial ratios using direct surface, digital, and ML methods. The face detection algorithm used in this paper was developed and distributed for open-access use by Google's ML Kit project. We found that ML measurements performed poorly in facial proportions that rely on intraocular distance, while performing better in measurements relying on nose, mouth, and face widths. Overall, none of the measurements calculated by our app reached an acceptable level of agreement with either digital or direct surface methods, as indicated by ICC values. The poor performance could be attributed to several factors in the development of these algorithms. In the data preparation stage, manually labeled landmark positions may have not been accurate enough for use in facial analysis, creating ratios different than the ground truth. Bias may have been introduced either due to homogenous data or inadequate regularization during the training phase. Lastly, the algorithm appeared biased toward facial symmetry and contours that increase proportionally. This was evident in the analysis of faces with hypertelorism or facial droop where the app would artificially lengthen the eye width to match larger intercanthal distances or correct for the droop, respectively.

Machine learning algorithms have potential clinical and research applications within the field of plastic surgery by automating and streamlining data extraction and analysis of clinically important data.^18,19^ Convolutional neural networks (CNNs), a powerful deep learning network architecture used in computer vision applications, form the backbone of most modern approaches to facial recognition.^12,20^ CNNs are increasingly used in medical diagnostics where fast and repetitive pattern recognition is needed.^21^ Barriers to the development of these algorithms go beyond knowledge expertise in the field of computer vision. To train a feature extraction model such as a CNN, researchers must first acquire a sizeable database (n>>1000) of images, manually label landmarks of interest, and have access to expensive and energy-intensive computing resources for training and validation of their models.^22,23^

Successful applications of ML in plastic surgery include accurate diagnosis and prognosis of burn patients, identification of congenital facial deformities, and various cosmetic surgery assessment applications.^24^ Currently, however, the majority of published literature in facial recognition algorithms and plastic surgery has focused on how the performance of preexisting facial recognition algorithms is impacted by patients who have undergone plastic surgery procedures.^12,14^ For example, an ML algorithm designed to predict an individual's gender has been shown to be more likely to predict a feminine gender in a patient after facial feminization surgery and this has been qualified as an indicator of successful facial feminization surgery.^25^ Although certainly interesting to experiment with, many research projects exploring ML in plastic surgery do not offer or discuss strategies for clinical translation. For clinicians to begin using ML solutions, barriers in the development process from model training to deployment and validation must be addressed. In this study, we examined the utility of a premade facial landmark detection algorithm for performing facial analysis using a smartphone camera application for a potential scalable pathway for clinical adoption.

Automatic facial feature analysis has been previously described in the literature. For facial paralysis assessments, *Facegram*, Facial Assessment by Computer Evaluation (FACE), and auto-eFACE are ML-based computer software tools capable of analyzing static or dynamic facial measurements with high accuracy.^26–28^ Although they require photography equipment and data transfer to a computer, they demonstrated high sensitivity and specificity in the assessment of facial paralysis before and after reanimation surgery. For cosmetic analysis, one study has demonstrated high accuracy for objective classification of female facial beauty using an algorithm based on decision trees.^29^ This study primarily focused on vertical proportions, though values were not compared to digital or surface measurements and the authors did not discuss clinical translation. Aarabi et al proposed an automatic facial feature extraction procedure based on data from 12 judges and a k-nearest neighbor classification.^30^ Their method, however, only provides coarse classification into 4 classes of beauty from which real-world applicability is difficult to appreciate. More recently, a deep learning algorithm (RhinoNet) was able to classify the rhinoplasty status of pre/post-operative patients and performed with effectively equivalent accuracy compared to plastic surgery residents.^31^ Although the algorithm is limited in utility, the authors successfully deployed RhinoNet on a smartphone application for use in clinical settings. Knoops et al and Mendoza et al developed robust machine learning algorithms capable of diagnostics, risk stratification, and treatment simulation for orthognathic surgery.^9,32^ As these models rely on 3D scans from CT or magnetic resonance imaging, they are beneficial for a narrow range of patient populations. Geisler et al. created a labeled database of craniofacial syndrome images and developed a CNN to classify anomalies based on images only.^33^ They were able to achieve a high level of sensitivity and specificity in classifying head shapes as metopic, sagittal, and unicoronal. To our knowledge, the tools mentioned above have not been widely deployed in clinical practice and there remains a need for a fast, versatile, and easily deployable automatic facial analysis tool.

The face detection model from ML Kit has several notable limitations. This study focused on facial proportions and not lengths as calibration of pixel to millimeter conversion requires fiduciary markers or complex depth calculation techniques. This model does not capture the forehead, therefore, it is not possible to calculate vertical proportions that include face height. A nasal analysis is particularly important for the rhinoplasty preoperative assessments.^34^ In this model, the nose consists of only 5 landmarks, far from enough to analyze the complex anatomy of nasal features. Additionally, this model cannot recognize facial landmarks in profile angles, limiting its use in measuring ear and nose proportions. The authors are unaware of any algorithms reported in the literature capable of analyzing profile views of human faces. Lastly, ML models contain bias from their training data. Algorithms may be biased against categories of race, gender, culture, and age if the original training dataset did not adequately represent those categories.^35^ As these data likely did not contain photos of cranial dysmorphology, the algorithm often attempts to infer symmetry where symmetry does not exist.

Although measurements calculated by the ML Kit algorithm did not reach a level of agreement with direct or digital methods, this study provides a paradigm for clinicians and researchers to assess the clinical utility and translation of open-access ML algorithms in plastic surgery. Additionally, Facekit is built with a modular design that supports the substitution of different algorithms with the potential for better performance on facial analysis tasks.

Future work will be required to improve the accuracy of open-access facial recognition software for its use in clinical practice. Specifically, these algorithms require more landmarks and an ability to function in both frontal and profile views. When investigators build high-performing algorithms, more effort should be made to making those accessible via smartphone or web-based interfaces for wider deployment and further validation. Our next step will be to assess the performance of a wider range of state-of-the art algorithms such as those built by Facebook, Microsoft, Amazon, and Apple to generate an index of clinical utility, as well as develop a novel algorithm based on up-to-date concepts in computer vision for specific use in plastic surgery assessments and provide wide deployment.

In conclusion, our study built and deployed a smartphone camera application for rapid facial feature extraction and analysis using a premade algorithm built by Google considered to be “best in class.” The performance of this algorithm was overall poor compared to direct surface and digital measurements. We conclude that pretrained facial recognition models are not sufficiently accurate to conduct a clinically useful facial analysis. Custom models trained with carefully labeled landmarks important to the plastic surgeon may offer a better solution to future automation of this task.

## References

[1] Edler R Agarwal P Wertheim D Greenhill D . The use of anthropometric proportion indices in the measurement of facial attractiveness. Eur J Orthod. 2006;28(3):274-281. doi:10.1093/ejo/cji098 16415084

[2] Bashour M . History and current concepts in the analysis of facial attractiveness. Plast Reconstr Surg. 2006;118(3):741-756. doi:10.1097/01.prs.0000233051.61512.65 16932186

[3] Schultz RC . Anthropometric facial proportions in medicine. JAMA. 1987;258(9):1245. doi:10.1001/jama.1987.03400090129051

[4] Farkas LG Bryson W Klotz J . Is photogrammetry of the face reliable? Plast Reconstr Surg. 1980;66(3):346-355. doi:10.1097/00006534-198066030-00004 7422721

[5] Farkas LG Katic MJ Forrest CR , et al. International anthropometric study of facial morphology in various ethnic groups/races. J Craniofac Surg. 2005;16(4):615-646. doi:10.1097/01.SCS.0000171847.58031.9E 16077306

[6] Farkas LG Hreczko TA Kolar JC Munro IR . Vertical and horizontal proportions of the face in young adult North American Caucasians: revision of neoclassical canons. Plast Reconstr Surg. 1985;75(3):328-338. doi:10.1097/00006534-198503000-00005 3883374

[7] Farkas LG Katic MJ Forrest CR . Comparison of craniofacial measurements of young adult African-American and North American white males and females. Ann Plast Surg. 2007;59(6):692-698. doi:10.1097/01.SAP.0000258954.55068.B4 18046155

[8] Grauer D Cevidanes LSH Styner MA , et al. Accuracy and landmark error calculation using cone-beam computed tomography-generated cephalograms. Angle Orthod. 2010;80(2):286-294. doi:10.2319/030909-135.1 19905853 PMC2914273

[9] Mendoza CS Safdar N Okada K Myers E Rogers GF Linguraru MG . Personalized assessment of craniosynostosis via statistical shape modeling. Med Image Anal. 2014;18(4):635-646. doi:10.1016/j.media.2014.02.008 24713202

[10] Plooij JM Swennen GRJ Rangel FA , et al. Evaluation of reproducibility and reliability of 3D soft tissue analysis using 3D stereophotogrammetry. Int J Oral Maxillofac Surg. 2009;38(3):267-273. doi:10.1016/j.ijom.2008.12.009 19167191

[11] Lee S . Three-dimensional photography and its application to facial plastic surgery. Arch Facial Plast Surg. 2004;6(6):410-414. doi:10.1001/archfaci.6.6.410 15545536

[12] Zuo KJ Saun TJ Forrest CR . Facial recognition technology: a primer for plastic surgeons. Plast Reconstr Surg. 2019;143(6):1298e-1306e. doi:10.1097/PRS.0000000000005673 31136498

[13] Jarvis T Thornburg D Rebecca AM Teven CM . Artificial intelligence in plastic surgery: current applications, future directions, and ethical implications. Plast Reconstr Surg Glob Open. 2020;8(10):e3200. doi:10.1097/GOX.0000000000003200 PMC7647513 33173702

[14] Bouguila J Khochtali H . Facial plastic surgery and face recognition algorithms: interaction and challenges. A scoping review and future directions. J Stomatol Oral Maxillofac Surg. 2020;121(6):696-703. doi:10.1016/J.JORMAS.2020.06.007 32574869

[15] Mateen BA Liley J Denniston AK Holmes CC Vollmer SJ . Improving the quality of machine learning in health applications and clinical research. Nat Mach Intell. 2020;2(10):554-556. doi:10.1038/s42256-020-00239-1

[16] ML Kit | Google Developers. https://developers.google.com/ml-kit/guides

[17] Pixel 5 tech specs – Google Store. https://store.google.com/ca/product/pixel_5_specs?hl=en-GB. Accessed August 10, 2021.

[18] Liang X Yang X Yin S , et al. Artificial intelligence in plastic surgery: applications and challenges. Aesthetic Plast Surg. 2020;45(2):784-790. doi:10.1007/S00266-019-01592-2 31897624

[19] Kanevsky J Corban J Gaster R Kanevsky A Lin S Gilardino M . Big data and machine learning in plastic surgery: a new frontier in surgical innovation. Plast Reconstr Surg. 2016;137(5):890e-897e. doi:10.1097/PRS.0000000000002088 27119951

[20] Adjabi I Ouahabi A Benzaoui A Taleb-Ahmed A . Past, present, and future of face recognition: a review. Electronics. 2020;9(8):1188. doi:10.3390/electronics9081188

[21] Esteva A Chou K Yeung S , et al. Deep learning-enabled medical computer vision. NPJ Digit Med. 2021;4(1):1-9. doi:10.1038/s41746-020-00376-2 33420381 PMC7794558

[22] Algorithms for Image Processing and Computer Vision – J. R. Parker – Google Books.

[23] García-Martín E Rodrigues CF Riley G Grahn H . Estimation of energy consumption in machine learning. J Parallel Distrib Comput. 2019;134:75-88. doi:10.1016/J.JPDC.2019.07.007

[24] Mantelakis A Assael Y Sorooshian P Khajuria A . Machine learning demonstrates high accuracy for disease diagnosis and prognosis in plastic surgery. Plast Reconstr Surg Glob Open. 2021;9(6):e3638. doi:10.1097/GOX.0000000000003638 PMC8225366 34235035

[25] Zuo K Forrest C . Facial recognition neural networks confirm success of facial feminization surgery. Plast Reconstr Surg. 2021;147(2):354E-355E. doi:10.1097/PRS.0000000000007562 33177465

[26] Gerós A Horta R Aguiar P . Facegram—objective quantitative analysis in facial reconstructive surgery. J Biomed Inform. 2016;61:1-9. doi:10.1016/J.JBI.2016.03.011 26994664

[27] Hadlock TA Urban LS . Toward a universal, automated facial measurement tool in facial reanimation. Arch Facial Plast Surg. 2012;14(4):277-282. doi:10.1001/ARCHFACIAL.2012.111 22508895

[28] Miller MQ Hadlock TA Fortier E Guarin DL . The auto-eFACE: machine learning-enhanced program yields automated facial palsy assessment tool. Plast Reconstr Surg. 2021;147(2):467-474. doi:10.1097/PRS.0000000000007572 33235050

[29] Gunes H Piccardi M . Assessing facial beauty through proportion analysis by image processing and supervised learning. Int J Hum Comput Stud. 2006;64(12):1184-1199. doi:10.1016/J.IJHCS.2006.07.004

[30] Aarabi P Hughes D Mohajer K Emami M . The automatic measurement of facial beauty. Proc IEEE Int Conf Syst Man Cybern. 2001;4:2644-2647. doi:10.1109/ICSMC.2001.972963

[31] Borsting E Desimone R Ascha M Ascha M . Applied deep learning in plastic surgery: classifying rhinoplasty with a mobile app. J Craniofac Surg. 2020;31(1):102-106. doi:10.1097/SCS.0000000000005905 31633665

[32] Knoops PGM Papaioannou A Borghi A , et al. A machine learning framework for automated diagnosis and computer-assisted planning in plastic and reconstructive surgery. Sci Rep. 2019;9(1):1-12. doi:10.1038/s41598-019-49506-1 31537815 PMC6753131

[33] Geisler EL Agarwal S Hallac RR Daescu O Kane AA . A role for artificial intelligence in the classification of craniofacial anomalies. J Craniofac Surg. 2021;32(3):967-969. doi:10.1097/SCS.0000000000007369 33405463

[34] Tollefson TT Sykes JM . Computer imaging software for profile photograph analysis. Arch Facial Plast Surg. 2007;9(2):113-119. doi:10.1001/archfaci.9.2.113 17372065

[35] Khalil A Ahmed SG Khattak AM Al-Qirim N . Investigating bias in facial analysis systems: a systematic review. IEEE Access. 2020;8:130751-130761. doi:10.1109/ACCESS.2020.3006051

